# Targeting NLRP3 signaling with a novel sulfonylurea compound for the treatment of vascular cognitive impairment and dementia

**DOI:** 10.21203/rs.3.rs-5611378/v1

**Published:** 2024-12-20

**Authors:** Adnan Akif, Thi Thanh My Nguyen, Langni Liu, Xiaotian Xu, Amol Kulkarni, Jianxiong Jiang, Yang Zhang, Jiukuan Hao

**Affiliations:** University of Houston; University of Houston; The University of Texas Health Science Center at Houston; The Affiliated Hospital of Yangzhou University; The University of Texas at El Paso; University of Tennessee Health Science Center; University of Houston; University of Houston

**Keywords:** Inflammasome, Inflammation, Ischemia, NLRP3, Vascular Dementia

## Abstract

**Background:**

As a key inflammatory factor, the nucleotide-binding oligomerization domain (NOD)-like receptor protein 3 (NLRP3) inflammasome plays a crucial role in neuroinflammation and the progression of neurodegenerative diseases. Dysregulation of NLRP3 signaling can trigger various inflammatory responses in the brain, contributing to the development of neurodegenerative diseases such as ischemic stroke, vascular dementia (VaD), Alzheimer’s disease (AD), Parkinson’s disease (PD), and amyotrophic lateral sclerosis (ALS). Therefore, the NLRP3 signaling pathway is a promising therapeutic target for the treatment of neurodegenerative diseases, including VaD.

**Methods:**

In this study, we investigated the therapeutic effects of a synthetic sulfonylurea NLRP3 inhibitor, AMS-17, in a VaD mouse model using bilateral common carotid artery stenosis (BCAS) and elucidated the underlying mechanisms. All mice were randomly divided into three groups: Sham, VaD + Vehicle, and VaD + AMS-17. Cognitive function was assessed using the Y-maze and Morris water maze (MWM) on the 50^th^ day after BCAS. Brain sections and blood serum samples were collected for biomarker analysis and immunohistochemistry. Neurodegeneration, expressions of the molecules involved in the NLRP3 signaling pathways, tight junction proteins, and myelination were assessed using western blotting and immunofluorescence (IF). The levels of Interleukin-1 beta (IL-1β), Tumor Necrosis Factor-alpha (TNF-α) and Interleukin-4 (IL-4) in the blood were measured using ELISA.

**Results:**

AMS-17 treatment improved cognitive function, enhanced blood-brain barrier (BBB) integrity, and promoted remyelination in VaD mice. Additionally, AMS-17 reduced neurodegeneration and decreased the expression of NLRP3 and its associated proteins, Apoptosis-associated speck-like protein (ASC), and cleaved caspase-1 in the brain. It also lowered pro-inflammatory TNF-α and IL-1β levels, while increasing the anti-inflammatory IL-4 level in the blood.

**Conclusions:**

The findings of this study provide the first promising evidence for the use of AMS-17 in VaD treatment in mice. This study introduces AMS-17 as a novel chemical scaffold with NLRP3 inhibitory activity, which can be further developed for the treatment of VaD in humans.

## Introduction

Vascular dementia (VaD) is the second most common form of dementia after Alzheimer’s disease among Alzheimer’s disease and related dementias (ADRD). VaD is caused by cerebral vascular diseases, hypertension, chronic cerebral hypoperfusion, cerebral ischemia, and post-ischemic inflammation. It also has the highest mortality rate among all forms of dementia. A large cohort study conducted in the U.S. found that, on average, Alzheimer’s patients live up to 7.1 years after diagnosis, whereas VaD patients only live an average of 3.9 years (1). This lower survival rate in VaD may be due to co-existing vascular conditions, such as ischemic and hemorrhagic events. Cerebral ischemic injury leads to neuronal death through various mechanisms, with inflammation being a key contributor to neurodegeneration in VaD. The role of inflammation in VaD is becoming increasingly recognized, as numerous inflammatory cytokines are aberrantly elevated in the brain during VaD (2). Therefore, targeting neuroinflammatory responses is essential for developing effective treatments for VaD. Despite significant efforts, a clinically effective drug for VaD treatment has yet to be identified. However, much effort has not yielded a clinically useful candidate drug for VaD treatment. Development of effective therapeutics for ADRD represents an urgent and unmet challenge

The NLRP3-mediated microglial inflammation plays a crucial role in the pathophysiology of VaD. Elevated NLRP3 expression in microglia is implicated in brain injury, neurodegenerative, and neuroinflammatory disorders (3,4). NLRP3 activation stimulates caspase-1 activity, leading to the maturation and release of pro-inflammatory cytokine IL-1β (5,6). IL-1β then triggers the expression of inducible nitric oxide synthase (iNOS), further promoting microglial activation, cell injury, and death. Increased amyloid-β (Aβ) level in VaD serves as danger-associated molecular patterns (DMAPs) that activate NLRP3. Hypoperfusion and the resulting hypoxia in VaD are also shown to promote Aβ production (2). Chronic Aβ deposition in the brain, particularly in the hippocampus, leads to Toll-like receptor-4 (TLR4)-mediated microglial activation, upregulating pro-inflammatory cytokines such as TNF-α, IL-1β, Interleukin-6 (IL-6), and Interleukin-18 (IL-18), and contributing to neuronal death (7–9). Inhibiting NLRP3-mediated inflammation may provide a novel approach to treating neuroinflammatory diseases like VaD (4,10,11). Previous studies demonstrated the benefit of disrupting NLRP3 signaling in VaD; for example, Osthole, a natural product with NLRP3 inhibitory activity, was shown to reduce Aβ deposition in a rodent model of VaD (12). Additionally, MCC950, a small-molecule NLRP3 inhibitor, improved cognitive function and vascular integrity post-stroke (13). Together, these studies highlight NLRP3 inhibition as a promising strategy for ADRD treatment.

Moreover, NLRP3 activation also occurs in brain endothelial cells and affects the Blood Brain Barrier (BBB) function and integrity. The BBB, formed by endothelial cells with tight junctions, controls the movement of molecules and cells across it. BBB compromise in ADRD leads to inflammation and neuronal damage, with pathological changes in brain endothelial cells playing a critical role in ADRD progression. NLRP3 activation induces inflammation and oxidative stress in the brain’s vascular endothelium, contributing to endothelial dysfunction, tissue swelling, chronic inflammation, and thrombus formation in the brain. It has been shown that NLRP3-induced inflammation at the BBB causes brain endothelial cells injury and BBB damage (14–17). Furthermore, the reports have shown that inhibition of NLRP3 inflammasome alleviates hypoxic endothelial cell death and protects BBB integrity in murine stroke and traumatic brain injury models (14,17). Therefore, inhibiting brain endothelial inflammation is of clinical relevance for ADRD treatment. In addition, because the capillary fraction is only about 20%−30% of total brain tissue (18), the cerebral endothelial cell volume in the brain only represents about 1/1000 of the total brain volume. Therefore, if a drug distributes at the ratio of 4:1 between brain tissue beyond the BBB and endothelial cells, drug concentrations in endothelial cells may be 200-fold higher than the average concentration of whole brain. In other words, to achieve pharmacological effects in the BBB, much lower systemic drug doses should be sufficient when compared to that required for targeting neurons or glial cells. Therefore, inhibition of NLRP3 in brain endothelial cells will provide significant beneficial effects for the treatment of ADRD.

In summary, elevated NLRP3 expression in microglia and brain endothelial cells contributes to the pathogenesis of brain injury and neurodegenerative diseases like VaD. Targeting aberrant NLRP3 activation to inhibit inflammation has emerged as a novel therapeutic approach for VaD and other neurodegenerative disorders. The lead molecule in this study is expected to inhibit brain inflammation by targeting both microglia and the BBB, offering potentially greater efficacy than approaches targeting cells beyond the BBB.

Several compounds, such as isoliquiritigenin, resveratrol, and curcumin, have shown NLRP3 inhibitory activity (19–21). However, these natural products are unsuitable for drug development due to stability issues (22). To date, multiple BBB-penetrating small-molecule NLRP3 inhibitors, including RRx-001, Dapansutrile and ZYIL1 have successfully completed Phase II trial.(23–26). However, many of these candidates have drawbacks, such as renal failure and upper airway infections (26). No NLRP3 inhibitor has yet been successfully applied for dementia treatment. Therefore, it is crucial to find a novel molecule for NLRP3 inhibition for developing effective treatment of ADRD. In the search for novel NLRP3 inhibitors, we synthesized a collection of sulfonylurea compounds designed to eliminate the instability seen in their natural counterparts while maintaining a similar three-dimensional structure (27). The newly developed sulfonylurea compound, AMS-17, demonstrated inhibitory effects on NLRP3 inflammasome activity and inflammation in microglial cells (28). Our previous study showed that AMS-17 had anti-inflammatory effects by inhibiting expressions of NLRP3, its downstream components and cytokines, such as caspase-1, TNF-α, IL-1β and iNOS in microglial cells (28). In the present study, we further evaluated the efficacy of AMS-17 in improving cognitive function and its associated molecular changes in a mouse model of VaD to investigate the underlying mechanisms.

## Materials and methods

### Synthesis of AMS-17 and preparation of AMS-17 solution

AMS-17 was synthesized using a modification of our previously reported procedure for the synthesis of tertiary sulfonylurea compounds (Scheme 1) (Kulkarni et al. 2020). It began with the reaction of pyrimidin-5-amine with 3-chloropropyl isocyanate resulting in the formation of acyclic urea. The latter was subjected to deprotonation with excess sodium hydride to affect the intramolecular ring closure. Quenching the reaction with aryl sulfonyl chloride 5 afforded AMS-17 as a white solid. NMR (1H and 13C), FT-IR and ESI-MS mass spectra for the synthetic sample of AMS-17 were consistent with the proposed structure. For the preparation of the AMS-17 solution, 1 mg of AMS-17 powder was mixed with 1 mL of DMSO, yielding a stock concentration of 28.47 mM. This stock solution was subsequently diluted with 1X phosphate-buffered saline (PBS) to achieve the desired working concentrations.

### Experimental Animals and VaD Establishment

The study was carried out in compliance with ethical guidelines set by the University of Houston’s Institutional Animal and Use Committees (IACUC) and the National Research Council’s *Guide for the Care and Use of Laboratory Animals*. The University of Houston is fully accredited by the American Association for the Accreditation of Laboratory Animal Care (AAALAC). Briefly, 10-month-old male C57BL/6J mice were obtained from Charles River Laboratories, housed individually, and maintained on a 12-hour light cycle with food and water available ad libitum at 23°C. Mice were allowed to acclimate for at least one week before experimental procedures began. Mice were randomly assigned to three groups: Sham, VaD, and Treatment. The VaD group underwent bilateral common carotid artery stenosis (BCAS) surgery, involving the placement of microcoils around both common carotid arteries (29). The treatment group underwent the same BCAS procedure and was administered either 20 mg/kg AMS-17 or Vehicle for five consecutive days. The sham group underwent carotid artery exposure without stenosis.

### Y-Maze

**Figure 1:**
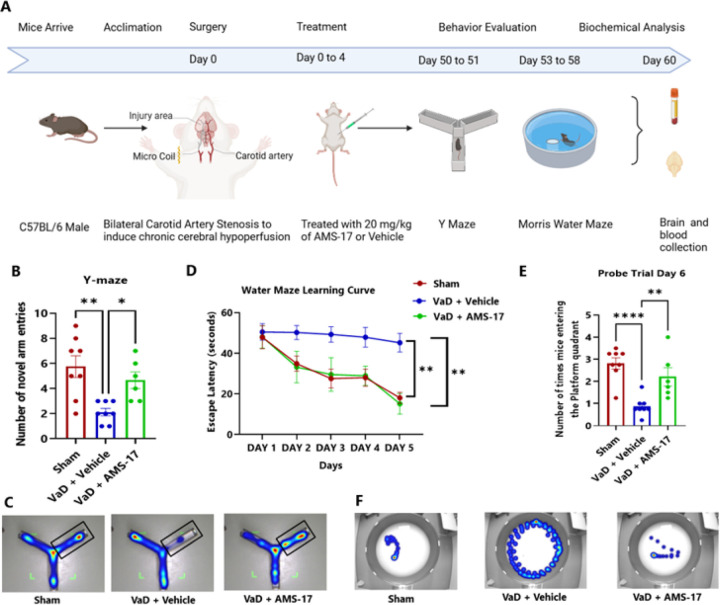
AMS-17 improves memory function in VaD mice.

The Y-maze spontaneous alternation performance test was conducted on the 50th day after BCAS (Fig. 1A) to assess novel environment recognition. The Y-maze apparatus consists of three identical arms spaced 120 degrees apart, designated as the start, novel, and other arms. The test comprised two phases: a training phase and a testing phase. During the 8-minute training phase, animals were allowed to explore two arms (start and other arms), while the novel arm was blocked. After a 1-hour interval, animals were placed in the start arm and allowed to explore all three arms for 8 minutes during the test phase. The number of entries into the novel arm was recorded. To prevent olfactory cues, the arms were cleaned with 70% ethanol between tests.

### Morris Water Maze

On the 53rd day after BCAS induction, mice underwent the Morris water maze test (Fig. 1A). This test consisted of a training session and a probe test session, during which the mice were placed in a circular swimming pool and allowed to explore for a maximum of 60 seconds. The test was conducted four times a day for five consecutive days, with the probe test taking place on the sixth day. The movements of the mice were tracked using Ethovision software, and the water temperature was kept around 25°C. A non-toxic white paint was added to the water to increase visibility, and the mice were dried with a towel and any fecal matter was removed from the water after each session.

### Samples Collection

After the final behavior tests on day 60 post-BCAS, brain samples were collected. The left hemisphere was stored at −80°C and cryosectioned for immunohistochemical analysis. The right hemisphere cortex was separated, solubilized in RIPA buffer, and homogenized with a Bead Blaster. Samples were centrifuged at 2000g for 15 minutes at 4°C, and supernatants were collected and stored at −20°C for Bradford assay and western blot analysis. Blood samples were also collected and centrifuged at 2000g and 4°C for 10 minutes to obtain serum for cytokine measurement.

### Immunofluorescence staining

Mouse brain tissues were fixed with 4% paraformaldehyde (PFA) in PBS at pH 7.4, cryosectioned into 8 µm slices, and permeabilized with 0.1% Triton-X 100 for 30 minutes. Brain slices were blocked with 5% bovine serum albumin (BSA) for 1 hour and incubated with primary antibodies (Supplementary Table 1) overnight at 4°C. Secondary antibody incubation followed the next day for 1 hour (Supplementary Table 3), after which slices were mounted with DAPI antifade media. TUNEL and Fluoro Jade C (FJC) staining were performed according to the instructions of manufacturer (TUNEL; Catalog #30063, Biotium, USA & Fluoro Jade C; Catalog TR-100-FJ, Biosensis, USA). Images were acquired using a Zeiss LSM 710 microscope.

### Analysis and quantitation of images

**Figure 2:**
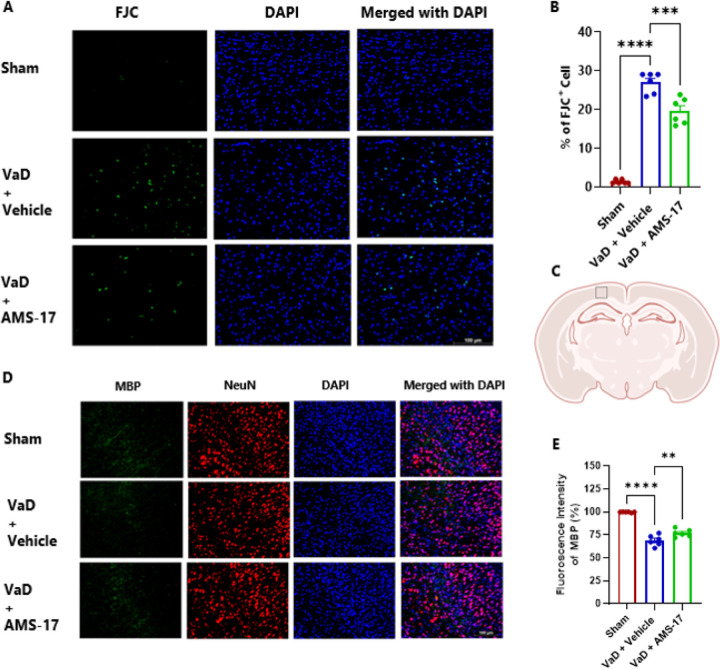
AMS-17 reduces neuron degeneration and demyelination in the cortical regions in VaD mice.

Images were acquired at consistent exposure times and analyzed using automated ImageJ (NIH) software. Following TIFF export and 8-bit conversion, images underwent edge detection, sharpening, thresholding, and water shedding. Normalization was achieved using ImageJ’s set scale function. Results presented as percentage of positive cells relative to total cells. Mean intensity analysis involved exporting images as TIFF files using standardized parameters. Images were imported into ImageJ, converted to 8-bit format, and analyzed using the measure function to calculate mean intensity. Results were expressed as relative integrated density (%) values relative to controls. For each mouse specimen, three cortical images from regions of interest (Fig. 2C) were acquired. All analyses were conducted in a blind fashion.

### Western Blotting

Protein concentrations were measured using a colorimetric Bradford assay kit (Biorad, USA), following the manufacturer’s protocol. Cortical lysates were solubilized in sodium dodecyl sulfate (SDS) sample buffer at 40 µg per lane and subjected to 10% SDS-polyacrylamide gel electrophoresis (SDS-PAGE) at 100 V for 110 minutes. Proteins were then transferred to polyvinylidene difluoride (PVDF) membranes, blocked with 5% BSA for 1 hour, and incubated with primary antibodies overnight at 4°C (Supplementary Table 2). Membranes were incubated with an HRP-conjugated secondary antibody for 1 hour (Goat anti-Rabbit IgG (H + L) Secondary Antibody, HRP, Invitrogen, CAT #31460) and visualized using Super Signal™ West Pico PLUS Chemiluminescent Substrate (Cell Signaling, USA). Bands were imaged using a Bio-Rad Chemidoc Touch imaging system, and band density was quantified using Image Lab 6.1 software (Bio-Rad, USA). Raw western blot bands are shown in the Supplementary file.

## ELISA

Cytokine concentrations (TNF-α, IL-1, and IL-4) in the blood were measured using ELISA kits. The TNF-α ELISA Kit (MTA00B-1) was obtained from R&D Systems, USA, while the IL-1 beta (BMS6002-2) and IL-4 (BMS613) ELISA kits were obtained from Invitrogen, USA. Briefly, the 96-well microplates were coated with a capture antibody overnight at 4°C and washed the wells 4 times, and then added a Diluent for 1 hour and washed the wells again. The plate was then incubated with antibodies for 2 hours, and washed 4 times afterwards, and incubated with streptavidin-HRP conjugate for 30 minutes. Finally, TMB solution was added for color development and the absorbance was measured at 450 nm.

## Statistical Analysis

Image analysis was performed using Fiji (ImageJ), an open-source image processing software (National Institutes of Health, Bethesda, MD, USA). Data are presented as means ± SD. Significant differences between and within multiple groups were examined using one-way and two-way ANOVA test, followed by Tukey’s multiple comparison test in Prism software (GraphPad, La Jolla, CA, USA). A p-value of < 0.05 was considered significant.

## Results

### AMS-17 improves cognitive impairment in a VaD mouse model.

The Y-Maze test assesses spatial memory and learning. During the testing phase, the number of entries into the novel arm was recorded. The mice in VaD with Vehicle group showed impaired memory function, with an average of 1.83 entries into the novel arm. In contrast, mice treated with AMS-17 showed significant improvement in learning and memory with an average of 4.66 entries into the novel arm, which was closer to the performance of the sham mice (5.75) (Fig. 1B). These results indicate that AMS-17 treatment improves learning and memory functions in mice subjected to VaD.

Furthermore, the Morris water maze (MWM) was performed to evaluate cognitive functions. The results indicated that mice in the sham and AMS-17-treated VaD groups learned to reach the platform significantly faster than those in VaD with Vehicle group during the training phase (Fig. 1D). The average escape latency in sham group decreased from 47.85 seconds on day 1 to 18.01 seconds on day 5. Similarly, in the AMS-17-treated VaD group, the average escape latency decreased from 48.02 seconds on day-1 to 15.09 seconds on day-5. However, the average escape latency of mice in VaD with Vehicle group did not change significantly during the 5-day training period, remaining at 45.16 seconds on day-5. A significant reduction in escape latency was observed in the sham group, indicating normal learning and memory functions, while the VaD group showed impaired learning and memory, as evidenced by a lack of significant decrease in escape latency. AMS-17 treatment significantly reduced escape latency in VaD mice, indicating a recovery of learning and memory functions (Fig. 1D). To further evaluate spatial memory, a 30-second spatial probe trial was conducted on day-6 following the training trial. The platform was removed from the pool, and the average frequency of mice entering the platform quadrant was recorded. The frequency of entering the platform quadrant (2.2) was significantly higher in the AMS-17-treated VaD group, closer to that of the sham mice (2.8), compared to the VaD with Vehicle group, which had a frequency of just 0.8 (Fig. 1E). The MWM results further indicate that AMS-17 treatment improves learning and memory functions in mice subject to VaD.

### AMS-17 protects against neurodegeneration and demyelination in a VaD mouse model.

We assessed the extent of neurodegeneration in the brains of VaD mice using Fluoro-Jade C (FJC) staining. The number of degenerating neurons (FJC-positive cells) in the brain cortex area was detected and compared across different groups. The results revealed a minimal percentage of FJC-positive cells in the cortical area of the brain in the sham group, at 1.3%. In contrast, this percentage increased to 26.94% in VaD with Vehicle group. Importantly, AMS-17 treatment significantly reduced the percentage of FJC-positive cells to 19.61% (Fig. 2A & B). The results of FJC staining demonstrate that AMS-17 treatment protects neurons from degeneration and exerts a neuroprotective effect in VaD conditions. Along with neurodegeneration, demyelination is a key pathological feature in the brain of VaD and contributes to cognitive impairment. To investigate this, we examined the expression of myelin basic protein (MBP) as a marker of myelin integrity and myelination. Our findings revealed that the average MBP intensity in the VaD with Vehicle group decreased to 68.99% compared to the sham group (Fig. 2D & E). However, treatment with AMS-17 significantly increased MBP level in VaD mice. The average MBP intensity in AMS-17 treated mice rose to 73.89% (Fig. 2D & E), indicating enhanced myelination in the brain. These findings indicate that AMS-17 has a potential role in suppressing demyelination and neurodegeneration in the brain.

### AMS-17 protects the BBB in VaD Mice.

**Figure 3:**
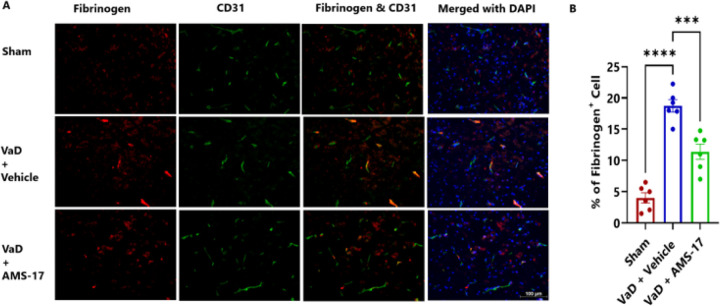
AMS-17 reduces fibrinogen deposition in the cortical regions of the brain in VaD mice.

**Figure 4:**
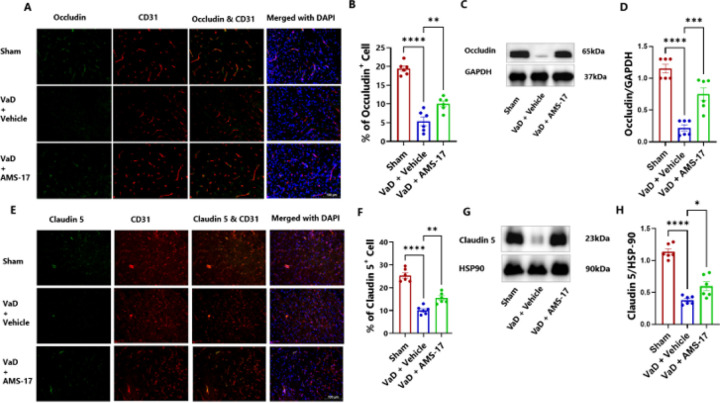
AMS-17 increases tight junction protein expressions in the cortical regions of the brain in VaD mice.

BBB integrity was evaluated by measuring fibrinogen deposition, a marker of BBB leakage, in the brain using immunostaining. We co-stained fibrinogen with CD31, a marker for endothelial cells, which line the blood vessels. The results showed that only 4.01% of cells were fibrinogen-positive in sham mice (Fig. 3A & B). In VaD with Vehicle group, the percentage of fibrinogen-positive CD31 cells increased to 18.72%, but AMS-17 administration lowered this to 11.38% (Fig. 3A & B). Additionally, we assessed the tight junction proteins, Occludin and claudin-5. Our study revealed that VaD induction significantly decreased occluding-positive CD31 cells from 19.42% (Sham) to 5.41% (VaD with Vehicle group). Importantly, AMS-17 treatment partially restored occludin expression to 10.04% (Fig. 4A & B). Consistently, western blot analysis of cortical lysates from the same mice showed that AMS-17 increased occludin expression from 0.22-fold in VaD with Vehicle group to 0.75-fold in the treatment group, relative to the sham group (Fig. 4C & D). A similar pattern was observed with claudin 5. As shown in Fig. 4C & D, the sham group had 25.42% claudin 5-positive CD31 cells, while in Vehicle-treated VaD group, the percentage significantly decreased to 10.16%. However, AMS-17 treatment increased claudin 5-positive CD31 cells to 15.44%. Western blot analysis further confirmed that claudin 5 expression decreased to 0.37-fold in VaD with Vehicle group compared to the sham group, but AMS-17 treatment restored claudin 5 expression to 0.59-fold (Fig. 4E & F). These findings indicate that AMS-17 reduced BBB leakage by promoting the expressions of tight junction, thereby helping maintain BBB integrity.

### AMS-17 suppresses expressions of NLRP3 and its associated proteins in cortex of the VaD mouse brain.

**Figure 5:**
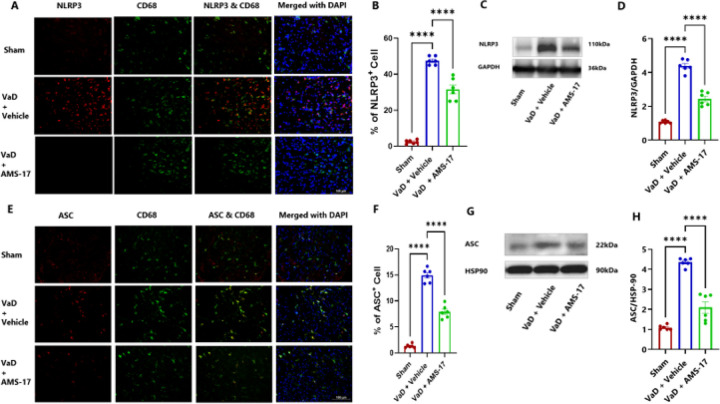
AMS-17 inhibits microglial NLRP3 and ASC expression in the cortical regions of the brain in VaD mice.

**Figure 6:**
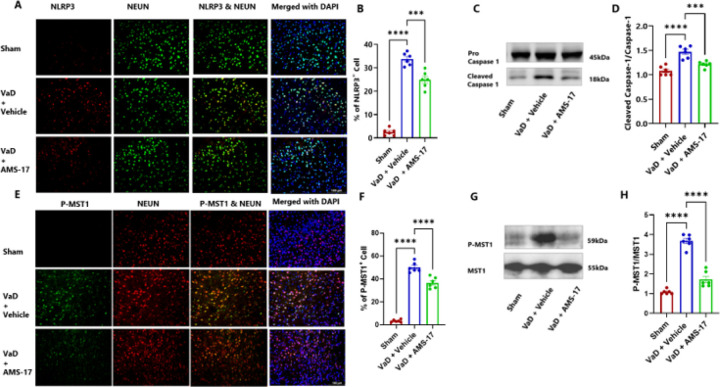
AMS-17 inhibits NLRP3, Cleaved caspase-1 and P-MST1 expression in the cortical regions of the brain in VaD mice.

To elucidate the underlying mechanism of AMS-17 in VaD treatment, we investigated its effect on the NLRP3 pathway. We hypothesized that AMS-17 inhibits NLRP3 and its associated proteins, ASC and cleaved caspase-1 expressions. To test this, we measured the protein levels of NLRP3, ASC, pro-caspase-1 and cleaved caspase-1 through western blotting of cortical brain homogenates and immunostaining of frozen brain sections collected on day 60 after VaD induction. As expected, NLRP3 was expressed in microglia, as indicated by double IF staining of NLRP3 with CD68. IF analysis of mouse brain cortex revealed that only 1.5% of microglia (CD68-positive cells) expressed NLRP3 in sham mice (Fig. 11A & B). In the Vehicle-treated VaD group, the percentage of microglia expressing NLRP3 increased to 47.21%, but treatment of AMS-17 lowered this to 31.58% (Fig. 5A & B). We also examined ASC and cleaved caspase 1 expression. VaD induction increased the expression of ASC-positive CD68 cells from 1.3% (Sham) to 14.91% (VaD with Vehicle), and AMS-17 reduced the expression of ASC-positive CD68 cells to 7.91% (Fig. 5C & D). Western blot analysis of mouse cortical lysates revealed that AMS-17 treatment significantly reduced the average expression of NLRP3 from 4.38-fold to 2.43-fold (Fig. 5C & D), ASC from 4.35-fold to 2.09-fold (Fig. 5G & H), and cleaved caspase-1 from 1.46-fold to 1.23-fold (Fig. 6C & D) in the AMS-17 treated VaD group compared to the VaD with Vehicle group (Fig. 5C, D, G & H). Interestingly, we observed NLRP3 expression in neurons, in line with prior research (30). Only 2.51% of neurons (Neu-N-positive cells) expressed NLRP3 in sham mice. But the percentage of NLRP3-positive neurons increased to 33.75% in VaD with Vehicle group, but the AMS-17 treatment reduced it to 24.91%. (Fig. 6A & B). We also investigated the molecular mechanism of AMS-17 by measuring the phosphorylation levels of MST1 in the cortex of mice using IF and western blotting. We co-stained P-MST1 with NeuN. Our staining results showed that the percentage of P-MST1-positive neurons increased to 49.23% in Vehicle-treated VaD group, while only 4.2% of neurons were P-MST1-positive in the sham group. AMS-17 treatment significantly reduced the percentage of P-MST1-positive neurons in VaD mice to 36.7% (Fig. 6E & F). Western blot data further confirmed these changes in P-MST1. Compared to the sham group, the expression of P-MST1 was 3.55-fold higher in the VaD with Vehicle group. Interestingly, AMS-17 treatment reduced the P-MST1 level to 1.72-fold (Fig. 6G & H). These findings indicate that AMS-17 treatment inhibits NLRP3 pathway activation and neuroinflammation in VaD, while also modulating the Hippo-MST1 signaling pathway to exert neuroprotective effects.

### AMS-17 treatment modulates inflammatory and anti-inflammatory cytokine level in VaD mice.

**Figure 7:**
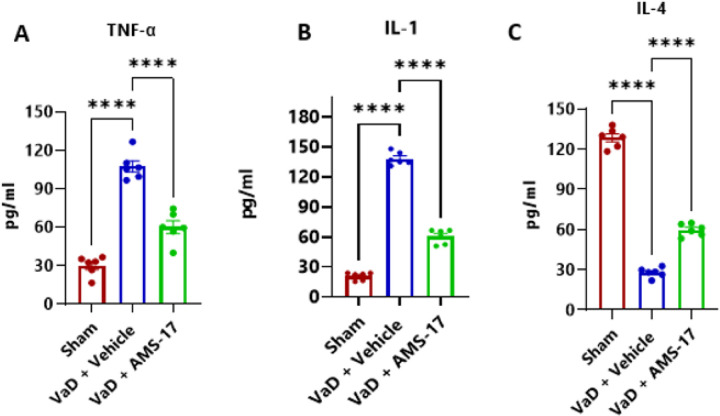
AMS-17 regulates cytokines release

We have assessed the levels of various cytokines, IL-1β, TNF-α (both downstream products of NLRP3 activation and key drivers of inflammation), IL-4 (a key anti-inflammatory cytokine) in the serum samples of mice using ELISA. Our findings revealed that TNF-α level was significantly elevated in the Vehicle-treated VaD group (138.39 pg/ml), showing a 6.6-fold increase compared to the sham group (20.94 pg/ml). AMS-17 treatment reduced TNF-α levels to 60.58 pg/ml, representing a 1.8-fold decrease compared to the Vehicle-treated VaD group, indicating significant attenuation of TNF-α expression (Fig. 7A). We also found that AMS-17 significantly reduced systemic IL-1β levels from 107.48 pg/ml in the Vehicle-treated VaD group to 59.57 pg/ml in the AMS-17-treated group, representing a 1.8-fold reduction (Fig. 7B). In addition to evaluating pro-inflammatory cytokines, we examined the levels of the anti-inflammatory cytokine IL-4 in the serum to better understand the immune response. Our results showed that AMS-17 treatment significantly increased IL-4 levels in VaD mice, boosting IL-4 level by 2.1-fold, from 27.86 pg/ml in the Vehicle-treated VaD group to 59.71 pg/ml in the AMS-17-treated group (Fig. 7C). Collectively, these data show that AMS-17 not only reduces pro-inflammatory cytokines but also promotes the production of the anti-inflammatory cytokine IL-4, exerting anti-inflammatory effects.

## Discussion

Targeting NLRP3 signaling pathway to inhibit brain inflammation could be effective for treating ADRD and neurodegenerative diseases Although some NLRP3 inhibitors have been tested in clinical trials for inflammatory diseases, none have been successful in treating ADRD. AMS-17, a novel synthetic sulfonylurea compound, was developed using pharmacophore modeling and computational chemistry, structurally derived from isoliquiritigenin (27). Our previous studies showed that AMS-17 reduced NLRP3 along with pro-inflammatory markers (caspase-1, IL-1β, and TNF-α) in N9 microglia cells and inhibited LPS-induced microglial activation in mouse brains (28). Based on these findings, we proposed that AMS-17 has therapeutic effects in VaD by inhibiting the NLRP3 pathway.

In this study, we first investigated if AMS-17 has therapeutic effects on cognitive functions in VaD mice induced by BCAS surgery, as described previously (29). The BCAS model, which induces chronic ischemia through bilateral common carotid artery, mimics VaD and impairs spatial memory and learning abilities, as evidenced by Y-Maze and MWM tests. However, AMS-17 treatment (20 mg/kg for 5 consecutive days) significantly improved cognitive functions in VaD mice (Fig. 1). Since cognitive improvement is associated with reduced neuronal death and degeneration, we further investigated histological and molecular evidence to elucidate the mechanism of action of AMS-17. As expected, FJC staining revealed that AMS-17 reduced the number of degenerating neurons in the cortex of VaD mice (Fig. 2A & B). AMS-17 also enhanced myelin repair in VaD mice, as shown by MBP expression recovery (Fig. 2D & E). In addition, AMS-17 treatment reduced BBB leakage and maintained BBB integrity by promoting the expression of tight junction proteins (Figs. 3 & 4). Having demonstrated therapeutic effects at the behavioral and histological levels, we further explored the molecular pathways involved in AMS-17’s protective effects. We observed upregulation of NLRP3 and its associated proteins, ASC and cleaved caspase-1, in the cortex of VaD mice. AMS-17 treatment effectively inhibited the expression of these proteins (Figs. 5 & 6). Although the initial understanding of the roles of NLRP3 signaling is in immune response cell, microglia, recent studies have expanded the knowledge of NLRP3 in neurons and other cell types in the brain suggesting a more widespread and complex role for NLRP3 in the brain (31). Peng’s group showed that NLRP3 can spread from microglia to nearby neurons and endothelial cells, perpetuating inflammation and damage. Our findings confirm that NLRP3 is expressed in both microglia (Fig. 5A & B) and neurons (Fig. 6A & B), and that AMS-17 decreases the expression of NLRP3, ASC, and cleaved caspase-1 (Figs. 5 & 6). Our results align with previous studies using the same VaD model, which showed activation of NLRP3, caspase-1, and IL-1β in the brain during VaD (32) (33).

Preclinical studies have robustly demonstrated that IL-1β and IL-18 are key drivers of the inflammatory response that occurs in the context of VaD (32,33). We hypothesized that the anti-inflammatory effect of AMS-17 is not only reflected in the brain, but also should be shown in peripheral tissue. Indeed, pro-inflammatory cytokines TNF-α and IL-1β were elevated in the blood of VaD mice, while anti-inflammatory cytokine IL-4 was significantly reduced compared to normal mice (Figure. 7). AMS-17 modulated cytokine levels in blood showing as a reduction of pro-inflammatory TNF-α and IL-1β level, while an increase of anti-inflammatory IL-4 level in blood (Figure. 7). These results suggest a potential disruption in the balance between pro- and anti-inflammatory responses in VaD. Remarkably, AMS-17 nullified these detrimental changes in pro- and anti-inflammatory cytokines and resumed the balance of pro- and anti-inflammatory cytokines resulting in protective effects on the brain.

The activation of NLRP3 leads to microglial activation, leukocyte recruitment, and BBB disruption (34,35). Consistent with previous findings (36,37), we observed BBB disruption characterized by decreased expression of tight junction proteins Occludin and claudin 5 and increased fibrinogen expression in VaD mice (Figs. 3 & 4). Furthermore, recent studies have revealed that fibrinogen plays a crucial role in activating the microglial NLRP3 inflammasome (38). The implications of this mechanistic study suggest that fibrinogen-driven NLRP3 activation contributes to BBB dysfunction. Recent studies have demonstrated that IL-1β, a key downstream effector of the NLRP3 signaling pathway and plays a crucial role in disrupting the integrity of the BBB (39) highlighting the importance of NLRP3 pathway in regulating BBB function. Suppressing NLRP3 activity by AMS-17 using in the present study has shown its protective effects on BBB integrity in VaD mice. In addition, accumulating evidence indicate that NLRP3 inflammasome plays important roles in demyelination after injury (40,41). IL-1β has been shown to hinder relocation of oligodendrocyte and increase myelin repair in VaD mice (42). On the contrary, reducing IL-1β secretion has been found to repair myelin formation remyelination in VaD mice model (33). Pharmacological inhibition of NLRP3 and caspase-1/IL-1β production has been shown to prevent myelin loss (43–45). Intriguingly, our findings revealed aberrant myelination in the cortical region of VaD brains indicating a disruption in normal brain function, and inhibition of NLRP3 by AMS-17 restored myelination and myelin repair (Figure. 2D & E).

The Hippo pathway plays a crucial role in neurodegenerative processes, particularly MST1 signaling, which is implicated in microglial activation following ischemic injury (46). While the Hippo pathway regulates NLRP3 activation in liver ischemia/reperfusion injury (47), its relationship with NLRP3 in the brain remains unexplored. Increase of NLRP3 activity and P-MST1 were observed in inflammatory and apoptotic conditions and lead to cell death (17,48). In the present study, our findings reveal there are increased levels of NLRP3 and P-MST1 expressions in cortical tissue, while MST1 levels remained unchanged in Vehicle treated VaD mice brain. AMS-17 treatment inhibits P-MST1 (Fig. 6) to protect neurons and BBB from injury. The results also imply potential crosstalk between NLRP3 and Hippo-MST1 pathway in VaD, highlighting the intricate mechanisms of this disease.

## Conclusion

In conclusion, AMS-17 was able to improve cognitive functions, reduce neuronal degeneration and death, mitigate damage, and promote myelin repair in a VaD mouse model. The therapeutic effects of AMS-17 are associated with inhibition of NLRP3 and Hippo pathways, as well as suppression of inflammation. The molecular mechanism of action involves AMS-17 inhibiting the NLRP3 and Hippo pathways, which suppresses inflammation and apoptosis induced by chronic ischemic injury. Further studies are needed to investigate the safety and pharmacokinetic profiles of AMS-17 in vivo and to elucidate its mechanism of action in greater detail. This study provides evidence supporting the potential therapeutic application of NLRP3 inhibitors for VaD, with AMS-17 emerging as a promising therapeutic candidate for its treatment.

## Supplementary Material

Supplement 1 Scheme 1 is available in the Supplementary Files section.

## Data Availability

All data generated or analyzed during this study are included in this published article.

## Abbreviations

NLRP3: nucleotide-binding oligomerization domain (NOD)-like receptor protein 3
VaD: vascular dementia
AD: Alzheimer’s disease
PD: Parkinson’s disease
ALS: amyotrophic lateral sclerosis
BCAS: bilateral common carotid artery stenosis
MWM: Morris water maze
IL-1β: Interleukin-1 beta
TNF-α: Tumor Necrosis Factor-alpha
IL-4: Interleukin-4
ELISA: enzyme-linked immunosorbent assay
BBB: blood-brain barrier
ASC: Apoptosis-associated speck-like protein
ADRD: Alzheimer’s disease and related dementias
US: the United States
CNS: central nervous system
VAD: Vascular contributions to cognitive impairment and dementia
iNOS: inducible nitric oxide synthase
Aβ: amyloid-β
DMAPs: danger-associated molecular patterns
TLR4: Toll-like receptor-4
IL-6: Interleukin-6
IL-18: Interleukin-18
NMR (^1^H and ^13^C): nuclear magnetic resonance (Proton and Carbon)
FT-IR: Fourier transform infrared spectroscopy
ESI-MS: electrospray ionization mass spectrometry
DMSO: dimethyl sulfoxide
PBS: phosphate-buffered saline
IACUC: Institutional Animal Care and Use Committees
AAALAC: American Association for the Accreditation of Laboratory Animal Care
RIPA: Radioimmunoprecipitation Assay
PFA: paraformaldehyde
BSA: bovine serum albumin
DAPI: 4′,6-diamidino-2-phenylindole
TUNEL: Terminal deoxynucleotidyl transferase dUTP nick-end labeling
SDS: sodium dodecyl sulfate
SDS-PAGE: SDS-polyacrylamide gel electrophoresis (
V: Voltage
mM: millimolar
PVDF: polyvinylidene difluoride
IgG (H+L): Immunoglobulin G (heavy chains + light chains)
HRP: Horseradish peroxidase
TMB: 3,3′,5,5′-Tetramethylbenzidine
SEM: standard error of the mean
ANOVA: Analysis of Variance
FJC: Fluro-Jade C
MBP: Myelin Basic Protein
CD68: Cluster of Differentiation 68
MST1: Mammalian Sterile 20-like kinase 1
P-MST1: phosphorylated Mammalian Sterile 20-like kinase 1
YAP: Yes-Associated Protein
YAP – TAZ: Yes-Associated Protein – transcriptional co-activator with PDZ binding motif
IF: Immunofluorescence
CD31: Cluster of differentiation 31
NeuN: Neuronal nuclear protein
HSP-90: Heat Shock Protein 90
GAPDH: Glyceraldehyde-3-phosphate dehydrogenase
