# Supplementary material for: Targeting NLRP3 signaling with a novel sulfonylurea compound for the treatment of vascular cognitive impairment and dementia
Source: Res Sq. 2024 Dec 20:rs.3.rs-5611378. Preprint. [Version 1] doi: 10.21203/rs.3.rs-5611378/v1 (PMC11702818; doi:10.21203/rs.3.rs-5611378/v1)
Supplement: Supplement 1 — Scheme 1 is available in the Supplementary Files section. [file NIHPPRS5611378v1-supplement-1.pdf]

Supplementary Files

This is a list of supplementary files associated with this preprint. Click to download.

- SupplementaryFile1.docx
- GraphicalAbstract.png
- Scheme1.docx
